# Cellular immunotherapy in melanoma: the next frontier in cancer treatment

**DOI:** 10.1186/s13046-026-03762-y

**Published:** 2026-06-18

**Authors:** Anna-Lena Wien, Angeliki Stamtsis-Datsi

**Affiliations:** Institute for Transplantation Diagnostics and Cell Therapeutics, Medical Faculty, University Hospital Düsseldorf, Heinrich-Heine-University Düsseldorf, Gebäude 14.88 Moorenstr. 5, Düsseldorf, 40225 Germany

**Keywords:** Immunotherapy, CAR T cells, Tumor-infiltrating Lymphocytes, CAR NK cells, Adoptive T cell transfer

## Abstract

Therapy-resistant melanoma remains a major clinical challenge despite advances with immune checkpoint inhibitors and targeted therapies, as most patients ultimately exhibit primary or acquired resistance and progress through multiple toxic treatment lines with limited durable benefit. This resistance reflects not only T-cell dysfunction but also a profound quantitative deficit of tumor-reactive lymphocytes, compounded by myeloid- and T_reg_-mediated immunosuppression, impaired antigen presentation, and tumor dedifferentiation programs generating a cold tumor microenvironment. Cellular immunotherapies aim to overcome these barriers by supplying or priming new tumor-reactive repertoires while reshaping the tumor microenvironment and restoring durable immunity. Dendritic-cell vaccines safely induce broad, long-lived responses but show modest activity as monotherapy in advanced disease. Tumor-infiltrating lymphocyte therapy directly replaces the missing tumor-specific compartment and has demonstrated clinically meaningful response rates in checkpoint-refractory melanoma, though at the cost of complex, intensive manufacturing and conditioning requirements. Genetically engineered TCR-T and CAR T/CAR NK therapies provide programmable specificity but remain constrained by antigen selection, toxicity risks, and limited persistence in solid tumors. NK-cell platforms offer HLA-independent, off-the-shelf potential but require further optimization for trafficking, persistence, and resistance to suppression. Overall, rational combinations - particularly DC-priming integrated with adoptive cell therapy - represent the most promising path to durable tumor control in treatment-refractory melanoma.

**Graphical Abstract:**

**Figure:**
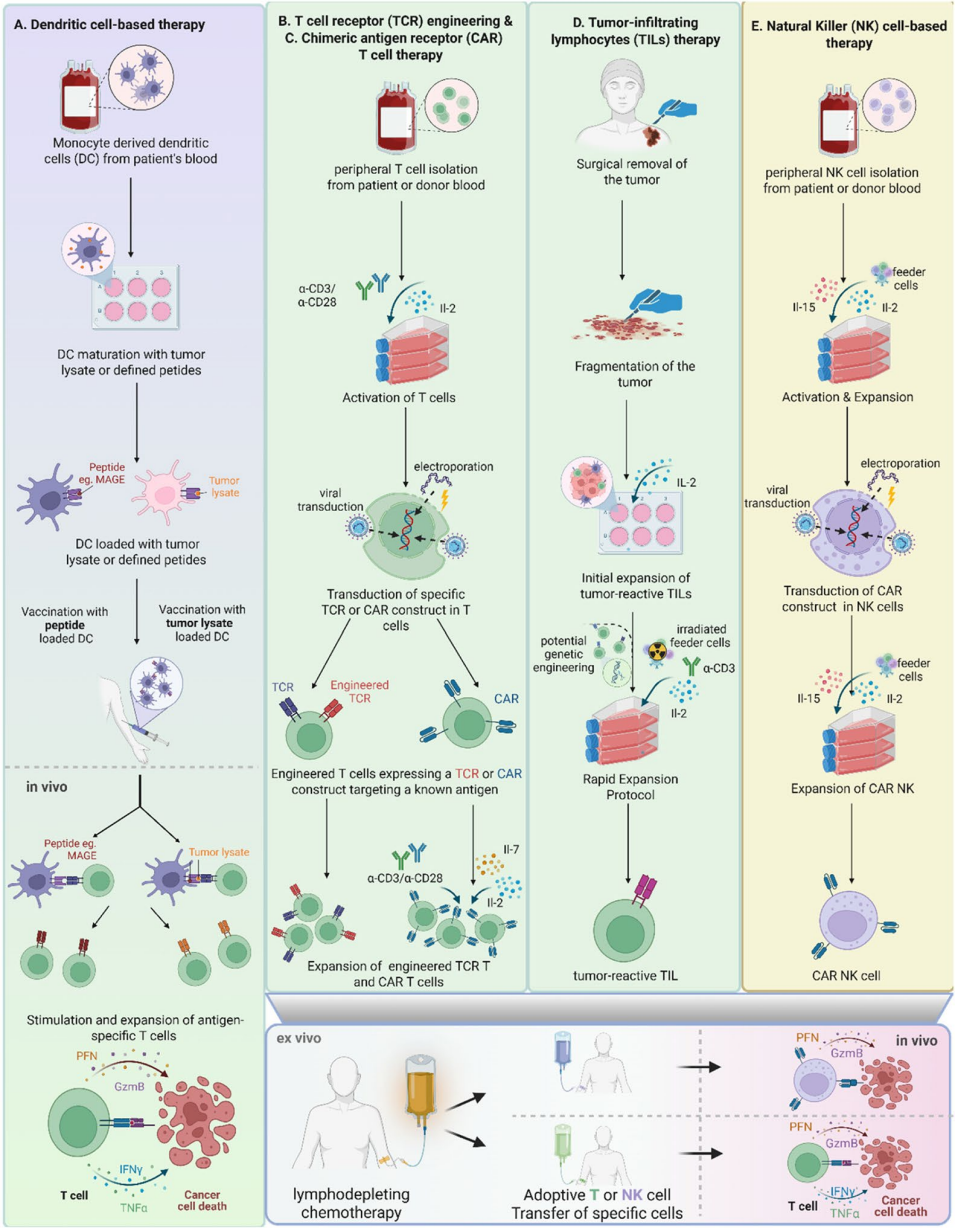
Overview of cellular immunotherapy platforms in melanoma and their core ex vivo manufacturing steps. (A) Dendritic cell-based vaccination uses monocyte-derived dendritic cells generated from patient blood, matured and loaded with defined tumor peptides or tumor lysate, and administered to prime antigen-specific T-cell responses in vivo. (B,C) TCR-T and CAR T-cell therapies start from peripheral blood T cells that are activated with anti-CD3 and anti-CD28, genetically modified by viral transduction or electroporation to express a tumor-specific TCR or CAR, expanded in culture, and infused after lymphodepleting chemotherapy to mediate antigen-directed tumor killing. (D) Tumor-infiltrating lymphocyte (TIL) therapy relies on surgical tumor resection, outgrowth of tumor-reactive TILs in IL-2, and rapid expansion using feeder cells and anti-CD3 prior to reinfusion after lymphodepletion. (E) NK cell-based therapy uses peripheral blood or cord blood NK cells expanded with IL-2 and IL-15 with optional feeder support and genetic engineering, including CAR expression, to generate cytotoxic products for adoptive transfer.

## Background

Melanoma is among the fastest-rising cancers and poses a disproportionate clinical burden. In 2022, an estimated 331,722 new cases occurred worldwide (age-standardized incidence 3.2 per 100,000) and 58,667 deaths [1], with the highest rates in fair-skinned populations and a decades-long upward trend in incidence, especially in Australia/New Zealand and North America [2] with an incidence rising of over 320% in the US since 1975. Arising from neural-crest-derived melanocytes, melanoma can develop in skin and at extra-cutaneous sites where melanocytes normally reside resulting in the epidermal-based cutaneous melanoma and the non-cutaneous melanomas, which occur in the uveal tract of the eye and on mucosal surfaces [3, 4].

Cutaneous melanoma is largely driven by ultraviolet (UV) exposure, resulting in a characteristic C > T mutational signature, high mutational burden, and frequent mitogen-activated protein kinase (MAPK) pathway alterations like mutations in B-Raf proto-oncogene (BRAF)^V600^ or Neuroblastoma RAS viral oncogene homolog (NRAS) [5]. In line with the central etiological role of UV exposure, more than 80% of global cutaneous melanoma cases in 2022 were estimated to be attributable to UV exposure [6]. By contrast, non-cutaneous melanomas usually lack UV signatures and arise through distinct genomic alterations [7, 8].

### Treatment regimens

These biologic distinctions have direct implications for clinical management and therefore shape both the initial work-up and subsequent therapy selection. The pathway begins with histologic confirmation and American Joint Committee on Cancer staging (I–IV) based on tumor features (T), lymph nodes (N), and metastases plus lactate dehydrogenase (LDH; M). In advanced cases, brain magnetic resonance imaging (MRI), LDH, and BRAF^V600^ testing guide prognosis and treatment selection [9]. Current international guidelines prioritize programmed cell death protein 1 (PD-1)-based immunotherapy as first-line for most patients regardless of BRAF status [10].

PD-1, cytotoxic T-lymphocyte-associated protein 4 (CTLA-4), and lymphocyte-activation gene 3 (LAG-3) are inhibitory immune checkpoint receptors on activated T cells that normally limit immune responses and maintain self-tolerance. Tumors exploit this system, for example by expressing Programmed death-ligand 1 (PD-L1), to suppress effector T-cell function and evade immune destruction. Monoclonal antibodies blocking PD-1, other checkpoint receptors, or their ligands release this brake, enhance tumor-specific T-cell activity, and can induce durable responses in some patients with advanced cancers, including melanoma [11–13].

In clinical practice, anti-PD-1 antibodies are established standard monotherapy options for advanced melanoma. This is supported by KEYNOTE-006, in which pembrolizumab significantly improved median overall survival (mOS) compared with ipilimumab (anti-CTLA-4; 32.7 vs. 15.9 months) [12]. Treatment therefore consists of pembrolizumab (anti-PD-1) or nivolumab (anti-PD-1) monotherapy, or dual-checkpoint blockade with nivolumab (anti-PD-1) plus ipilimumab or relatlimab (anti-LAG-3) [10]. Combination regimens further extend survival, reaching an mOS of 71.9 months with nivolumab plus ipilimumab in CheckMate-067 [14] and about 51.0 months with nivolumab plus relatlimab in RELATIVITY-047 [15].

For tumors harboring BRAF^V600^, combined BRAF inhibitor (BRAFi) and mitogen-activated protein kinase (MEK) inhibitor (MEKi) therapy remains a standard, rapidly acting option, achieving a median overall survival (mOS) of 25.9 months with dabrafenib (BRAFi) plus trametinib (MEKi) [16] and a five-year overall survival of about 35% with encorafenib (BRAFi) plus binimetinib (MEKi), rising to about 45% in patients with normal LDH levels [17]. Treatment selection between targeted therapy and immunotherapy is individualized [18], although randomized sequencing studies support starting with immunotherapy when clinically feasible [19].

Despite these advances, many patients with advanced metastatic melanoma (stage IV) still derive limited or no durable benefit from current systemic therapies. Approximately 40–55% show primary resistance to PD-1- or CTLA-4-based immune checkpoint inhibition (ICI), and about 25% of initial responders later develop acquired resistance [20, 21]. In addition, intensified regimens such as nivolumab plus ipilimumab cause grade 3–4 adverse events (AE) in around 60% of patients, often limiting treatment use or causing early discontinuation [22]. Together, these findings highlight persistent limitations of current checkpoint inhibition and targeted therapy, as many patients do not respond durably, relapse, or cannot tolerate full-intensity treatment [23].

Studies show this ceiling arises as chronic antigen exposure in melanoma drives CD8⁺ T cells into exhausted states with stable transcriptional and epigenetic changes and persistent inhibitory receptor expression [24]. This exhaustion is initially permissive to reversal by ICI. However, tumor-intrinsic alterations such as.

(I) loss of antigen presentation,
(II) defects in interferon-γ/Janus kinase-signal transducer and activator of transcription (JAK-STAT) signaling,
(III) oncogenic pathway activation,
(IV) tumor-extrinsic factors including T-cell exclusion,
(V) expansion of immunosuppressive immune cells including regulatory T cells (T_reg_) and myeloid-derived suppressor cells (MDSCs) as well as.
(VI) extensive dysfunction of the CD8⁺ compartment,

collectively drive primary and acquired resistance to PD-1, CTLA-4 and BRAF/MEK inhibition in melanoma [25–27]. Because ICI primarily reinvigorates pre-existing antitumor T-cell responses, its efficacy is favored by the presence of tumor-reactive lymphocytes and intact antigen presentation at baseline [13, 24, 28]. In cold or heavily pretreated tumors, however, low antigenicity and a myeloid-dominated, T cell-hostile microenvironment often leave too few potent tumor-specific T cells to be rescued by checkpoint blockade [24, 28]. Together, these findings identify a key biological and clinical gap: resistance to ICI may reflect not only inhibitory receptor signaling, but also insufficient quantity, specificity, or functional fitness of tumor-reactive lymphocytes within the tumor microenvironment [13, 23, 24, 28].

This unmet need provides a strong rationale for cellular therapies that directly supply highly tumor-reactive T cells or generate them ex vivo. Owing to its high mutational burden, frequent T-cell infiltration, and long-standing role as a model disease in immunotherapy, melanoma has been at the forefront of adoptive cell therapy development. Accordingly, a broader range of cellular approaches is now being pursued to exploit melanoma’s immunogenicity while overcoming key resistance mechanisms that limit antibody-based immunotherapy.

### Main Text

#### Cellular immunotherapies for melanoma

While immune checkpoint inhibitors and targeted therapies have transformed the prognosis of metastatic melanoma, many patients still develop resistance or cannot tolerate full-intensity regimens, creating a need for additional immune-based strategies [22, 25, 29]. Cellular immunotherapies seek to fill this gap by inducing de novo tumor-specific immunity or by supplying expanded and engineered effector cells able to persist and function in the tumor microenvironment. In melanoma, the most promising platforms include dendritic cell-based vaccines, tumor-infiltrating lymphocyte transfer, genetically engineered T-cell therapies such as T-cell receptor-engineered T cells (TCR-T) and chimeric antigen receptor (CAR) T cells, and natural killer (NK) cell-based approaches including CAR NK products [30, 31] (Graphical Abstract).

#### Dendritic-cell-based vaccines in melanoma

Dendritic-cell-based vaccination (DCV) in melanoma is a personalized immunotherapy in which autologous dendritic cells (DCs) are loaded ex vivo with tumor-associated antigens (TAAs), such as defined peptides or whole tumor lysates, to prime or boost tumor-specific CD4⁺ and CD8⁺ T-cell responses in vivo [32, 33]. As professional antigen-presenting cells (APCs), DCs efficiently process and present antigens on human leukocyte antigen (HLA) class I and II molecules and provide the co-stimulatory and cytokine signals required for effective T-cell activation and differentiation [34, 35].

#### Principle and manufacturing of DCV

In most melanoma protocols, dendritic cells (DCs) are generated from leukapheresis-derived monocytes cultured with granulocyte-macrophage colony-stimulating factor (GM-CSF) and interleukin-4 (IL-4). They are then matured using pro-inflammatory cytokine cocktails (containing tumor necrosis factor α (TNF-α), IL-1β, IL-6 and prostaglandin E2 (PGE2)) or toll-like receptor (TLR) agonists and loaded with melanoma-associated peptides, such as Melanoma antigen recognized by T cells 1 (MART-1), Glycoprotein 100 (gp100), tyrosinase, or Melanoma antigen gene (MAGE), or with autologous whole tumor lysate. The final product is typically administered repeatedly by intradermal, subcutaneous, or intranodal injection [34–36].

These products act as in vivo stimulators to elicit endogenous tumor-specific T cells and can be repeatedly administered in adjuvant and metastatic settings without lymphodepleting conditioning [33, 37]. This distinguishes DCV from adoptive T-cell transfer, as it aims to reshape the host T-cell repertoire rather than infuse large numbers of pre-expanded effector cells.

#### Clinical activity and safety

The clinical development of DC-based therapies in melanoma was pioneered by Nestle et al., who vaccinated metastatic melanoma patients with autologous monocyte-derived DCs (moDC) pulsed with defined melanoma peptides or autologous tumor lysate. This induced antigen-specific cytotoxic T lymphocytes, delayed-type hypersensitivity (DTH) responses, and objective tumor regressions in 5 of 16 evaluable patients, including 2 complete responses (CR) and 3 partial responses (PR), indicating clinical activity in a subset of patients. However, this small, non-randomized phase I study was not designed to assess OS or definitive clinical efficacy [32].

Subsequent phase I and phase II trials of peptide- or tumor lysate-pulsed mature DCs confirmed consistent immunogenicity, with vaccine-induced tumor-specific CD8⁺ T-cell responses detected in most evaluated patients. Akiyama et al. reported enzyme-linked immunospot (ELISPOT) responses against ≥ 2 melanoma peptides in 6 of 6 immunologically evaluable patients [38], while Ribas et al. detected tumor antigen-specific CD8⁺ T cells in 26 of 29 patients, with boosted or induced responses in 19 of 29 patients [36]. Despite this, objective responses remained modest, occurring in about 10% of patients with advanced melanoma, while 11–18% achieved prolonged stable disease [36, 38, 39]. These findings highlight the persistent gap between immunogenicity and clinical benefit, likely due to tumor burden, microenvironmental immunosuppression, and T-cell exhaustion [33, 35].

The toxicity profile of DCV is generally favorable and dominated by low-grade local and systemic AE. Most studies reported grade 1–2 flu-like symptoms, low-grade fever, fatigue, and transient injection-site reactions, whereas grade 3 toxicities were rare and treatment-related deaths were not observed [33, 36, 37]. Importantly, severe immune-mediated toxicities characteristic for ICI have not been documented for DCV, which makes these therapies particularly attractive for the adjuvant setting and for combination strategies in patients who have already received multiple lines of systemic treatment and allows a prolonged vaccination schedule [33, 37, 40].

#### Antigen formats and peptide-based strategies

Optimization of DC-based T-cell therapies in melanoma focuses on antigen selection and formulation to improve the quality, breadth, and durability of vaccine-primed T-cell responses [35, 41].

**Fig. 1:**
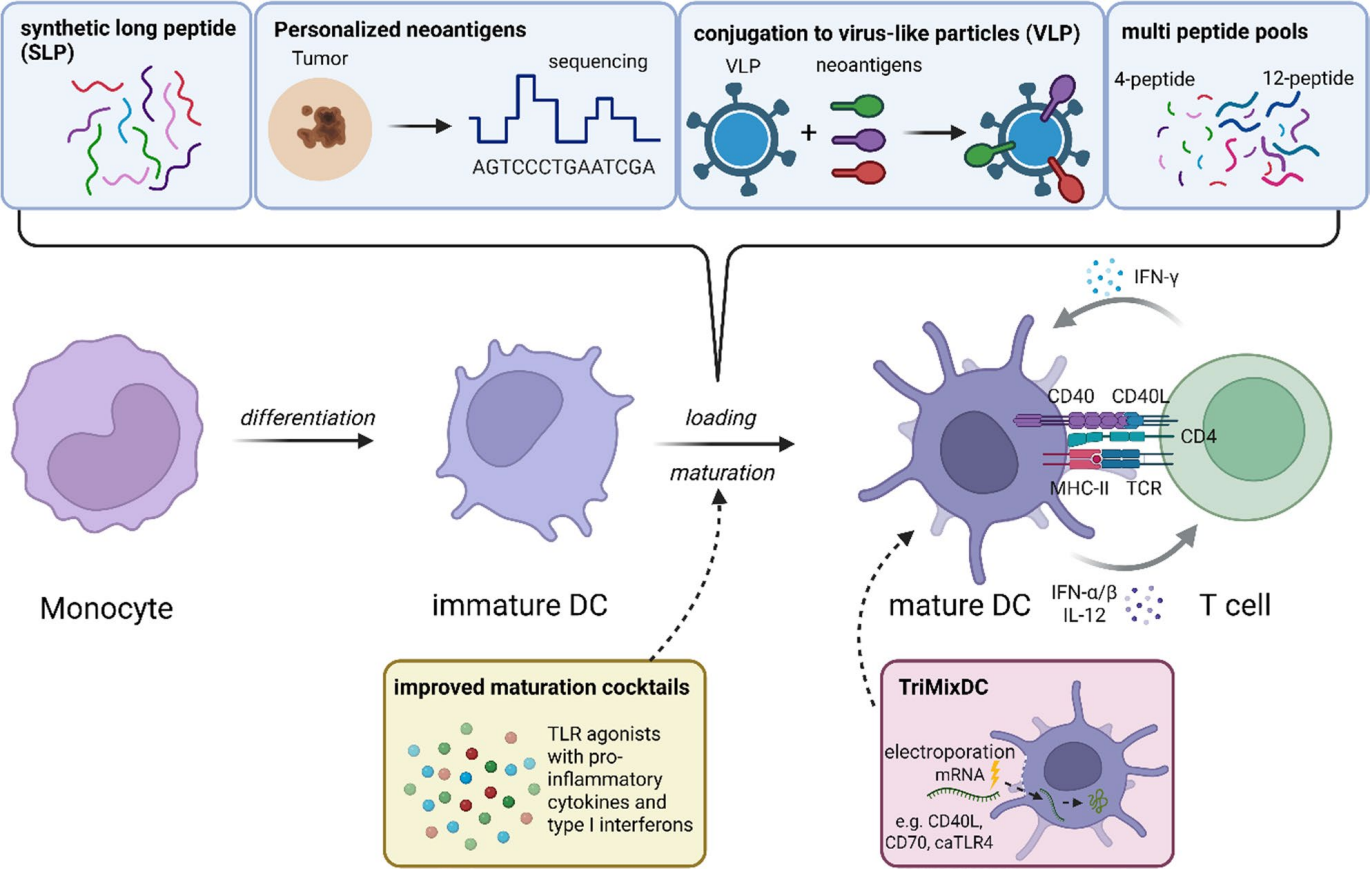
Advances in manufacturing of dendritic cell vaccination in melanoma. Autologous monocytes obtained from leukapheresis are differentiated ex vivo into monocyte-derived DCs. The resulting immature DCs are then matured and loaded in parallel with tumor-associated antigens, defined melanoma peptides, synthetic long peptides and patient-specific neoantigen peptides identified by tumor sequencing, or alternatively as autologous whole tumor lysate. Antigen formulation refinements, including conjugation to virus-like particles, encapsulation in nanoparticles, or the use of multi-peptide pools, can enhance antigen uptake and support efficient cross-presentation. Maturation is induced with TNF-α, IL-1β, IL-6, and PGE2, optionally complemented by toll-like receptor agonist and type I interferons. Third-generation approaches such as TriMix further potentiate maturation by electroporating moDCs with mRNA encoding CD40L, CD70, and constitutively active TLR4. The final mature, antigen-loaded DC product is administered by injection to stimulate endogenous tumor-specific CD4⁺ and CD8⁺ T-cell responses through antigen presentation on HLA class I and II, co-stimulation, and cytokine signaling

One approach ues synthetic long peptides (SLPs, Fig. 1), which require intracellular processing and cross-presentation and contain epitopes presented via HLA class I and class II, thereby engaging both CD8⁺ and CD4⁺ T cells more effectively than minimal epitope vaccines [42, 43]. Preclinical studies show that long-peptide-pulsed DCs achieve superior cross-presentation and enhanced activation of cytotoxic T cells (CTL) than short-peptide-loaded DCs [43, 44]. Personalized DCV incorporating patient-specific neoantigen peptides identified by tumor sequencing can broaden the targeted epitope repertoire and, in small melanoma cohorts, increase the breadth and diversity of circulating neoantigen-specific T-cell responses, although larger trials are still needed to establish clinical efficacy [45].

An alternative antigen-agnostic strategy is loading DCs with autologous whole tumor lysate, which includes shared differentiation antigens, cancer-testis antigens, and private or unknown neoantigens without predefined epitope selection [46]. In the phase I/II study by Salcedo et al. in stage III/IV melanoma, lysate-loaded autologous moDCs were feasible, safe, and immunogenic, with no grade 3–4 vaccine-related toxicities and enhanced T-cell responses to tumor lysate and TAA-derived peptides; among nine patients completing all vaccinations, four survived > 20 months, including one complete remission and one case of stable metastatic disease for ca. 10 months [46]. In the randomized phase III MIND-DC trial after complete resection of stage IIIB/C melanoma, intranodal vaccination induced functional tumor-specific T cells in about 67% of vaccinated patients versus 3.8% in the placebo arm, but did not significantly improve clinical outcome despite numerically longer 2-year progression-free survival (PFS) and OS [47]. Overall, tumor-lysate-loaded DC vaccines show biological activity and possible clinical benefit in selected patients, but the evidence remains limited, heterogeneous, and modest compared with contemporary adjuvant PD-1-based therapy.

Formulation refinements, including conjugation of tumor lysates to virus-like particles (Fig. 1) and encapsulation of tumor antigens with TLR agonists in nanoparticles, improve antigen uptake and cross-presentation. In preclinical melanoma and other solid-tumor models, these approaches elicit stronger CD8⁺ T-cell priming and better tumor control than conventional lysate-pulsed DCs [48, 49]. Likewise, TLR3 agonists such as poly I: C can generate more potent clinical-grade DCs; poly I: C-activated moDCs induce greater expansion of antigen-specific T cells and a lower Forkhead box P3 (FoxP3)⁺ T_reg_/T effector ratio than DCs matured with lipopolysaccharide or cytokine cocktails [50]. However, clinical validation in melanoma remains at an early stage.

#### Optimization of DC maturation and delivery

A second axis of optimization targets DC maturation and delivery route, as immature or incompletely matured DCs may induce tolerance, whereas fully matured DCs support effector and memory differentiation [51]. Maturation cocktails combining TLR agonists, pro-inflammatory cytokines, and type I interferons upregulate co-stimulatory molecules such as CD80, CD86, and CD40, enhance IL-12 production, and promote chemokine receptor expression favoring lymph node migration [52, 53]. In preclinical models and early human studies, these DCs induce stronger T helper type 1 (T_H_1) and cytotoxic T-cell (CTL) polarization than conventional GM-CSF/IL-4-derived DCs [53, 54].

The TriMix platform (Fig. 1) is a third-generation maturation strategy in which moDCs are electroporated with mRNA encoding CD40 ligand, CD70, and constitutively active TLR4, generating highly mature cells with enhanced T-cell stimulatory capacity and reduced donor-to-donor variability [55]. In pretreated advanced melanoma, TriMixDC-MEL was feasible and well tolerated and induced objective responses in about one quarter of patients, including durable complete responses [56]. In phase II, combination with the CTLA-4 antibody ipilimumab achieved an overall response rate of about 38%, with complete responses in about 20% and several responses lasting beyond two to three years [57]. These data suggest that optimized DC maturation can improve durable benefit, particularly in combination with checkpoint inhibition.

Alternative delivery routes for DC vaccines in melanoma should be viewed as route-optimization strategies rather than intrinsically superior approaches. Intradermal injection depends on active DC migration and is inefficient, with in vivo tracking showing that only up to 4% of injected DCs reach draining lymph nodes. Nevertheless, even small numbers of viable DCs in T-cell areas can induce antigen-specific responses [58]. Intranodal delivery is mechanistically attractive because it bypasses this migratory bottleneck, and an early randomized phase I melanoma trial reported superior CD8⁺ T-cell function after intranodal administration compared with intravenous or intradermal routes [59]. However, later comparative data did not confirm a general advantage: in 43 advanced melanoma patients, intradermal vaccination induced functional tumor-antigen-specific T cells more frequently than intranodal vaccination, despite greater nodal DC delivery [60]. Combined intradermal/intravenous approaches such as TriMixDC-MEL were safe and immunogenic in small phase Ib cohorts, but remain non-definitive [56]. Accordingly, the optimal route likely depends on DC maturation state, migratory capacity, antigen loading, and disease setting. This is underscored by the phase III MIND-DC trial, where intranodal natural DC vaccination induced functional antigen-specific T-cell responses but did not improve recurrence-free survival [47].

#### Defined melanoma peptides in DC and non-DC vaccines

A broad range of melanoma-associated peptides has been used to prime or boost T-cell responses in DC-based and conventional peptide vaccine trials. These include differentiation antigens such as Melan-A/MART-1, gp100, and tyrosinase, as well as cancer-testis antigens such as MAGE family members and New York esophageal squamous cell carcinoma 1 (NY-ESO-1), administered either as single epitopes or in peptide pools [61].

Single-epitope approaches, exemplified by the MAGE-3.A1 peptide DC vaccine, show that a shared tumor peptide can expand tumor-specific cytotoxic T cells and induce regression of individual metastases in a subset of patients, but responses are infrequent, often partial, and limited by antigen-loss escape [62]. By contrast, multi-peptide DC vaccines, including CD34-progenitor-derived DCs loaded with 4 HLA-A2-restricted melanoma peptides [63] moDCs pulsed with 5-peptide cocktails tailored to HLA-A2 or HLA-A24, consistently induce broader T-cell repertoires [64]. In these studies, most patients responded to at least one melanoma antigen and many to multiple epitopes, whereas no single peptide emerged as a dominant target across patients. Objective responses or disease stabilization occurred in a subset of patients, and broader epitope recognition often correlated with improved short-term disease control [63, 64].

Non-DC peptide vaccines provide complementary insight. In a randomized phase III trial in HLA-A2-positive advanced melanoma, addition of the gp100:209–217(210 M) peptide to high-dose IL-2 increased objective response rates (ORR) and prolonged PFS and OS compared with IL-2 alone, although absolute response rates remained modest [65]. Likewise, the NY-ESO-1 79–108 long-peptide vaccine with CpG-B induced integrated NY-ESO-1-specific CD8⁺ and CD4⁺ T-cell and antibody responses in virtually all evaluable patients, with durable polyfunctional cytokine production and epitope spreading, while clinical benefit mainly consisted of stable disease and occasional partial responses [66].

In the adjuvant setting, multi-peptide vaccines evaluated in the Mel39 and Mel44 programs were tested in completely resected high-risk melanoma (AJCC stage IIB-IV) arising from cutaneous, mucosal, or unknown primary sites [67, 68]. In Mel39, 4- and 12-peptide class I HLA-restricted vaccines induced high peripheral immunogenicity and were associated with long-term OS, with a non-significant trend favoring the 12-peptide arm [67] In the larger randomized phase II Mel44 trial, a 12-peptide vaccine plus melanoma-specific helper peptides (12MP+6MHP) showed more favorable long-term OS than the same vaccine plus tetanus helper peptide (12MP + tet) [68].

Overall, defined melanoma peptides such as MART-1, gp100, tyrosinase, MAGE-3, and NY-ESO-1 can induce strong antigen-specific T-cell responses in both DC-based and conventional peptide vaccines. Peptide pools more reliably broaden the cytotoxic T-cell repertoire and may improve outcomes within individual cohorts, but a clear survival advantage over single-peptide strategies has not been demonstrated in randomized melanoma trials (Table 1).

**Table 1:** Representative peptide formulations and their immunological and clinical readouts

Peptide or peptide set	Platform and clinical setting	Immunogenicity readouts	Clinical activity	Single-peptide vs. peptide-pool
MAGE-3.A1 minimal epitope (74)	Mature moDC + recall antigen in stage IV melanoma (*n* = 11).	MAGE-3.A1-specific CTL expansion in 8/11; recall responses boosted.	Metastatic lesion regression in 6/11; regressing lesions showed CD8⁺ infiltrates, whereas some non-regressing lesions lost MAGE-3.	A single shared peptide can induce tumor-specific CTLs and clinical regression, but responses were infrequent/partial; antigen-loss escape supports multi-epitope strategies.
Four-peptide HLA-A2 pool (MART-1, gp100, tyrosinase, MAGE-3) (81)	CD34⁺-progenitor-derived DCs pulsed with 4 HLA-A2-restricted melanoma peptides + Flu-MP/KLH in metastatic melanoma (*n* = 18).	Immune responses to KLH/Flu and ≥ 1 melanoma peptide in 16/18; 10/18 responded to > 2 antigens with T_H_1-polarized profiles.	Among 17 evaluable patients, ≥ 1 metastasis regressed in 7; responses to > 2 antigens were linked to less early progression.	Compared with a single peptide, a 4-peptide pool broadened immunity and was associated with better early disease control, although overall activity remained modest.
HLA-A2/A24 five-peptide DC cocktail (49)	Autologous moDC pulsed with 5 HLA-matched melanoma peptides + KLH in metastatic melanoma (phase I, *n* = 9).	All 6 immunologically evaluable patients developed CTL responses to > 2 epitopes; tetramer⁺ CD8 T cells were detected with a T_H_1-biased pattern.	Best responses: 1 CR, 1 PR, 1 SD, 6 PD.	A focused mid-sized peptide pool induced multi-specific CD8 responses and a small but notable objective response rate.
gp100:209–217(210 M) minimal epitope (77)	Soluble peptide vaccine + high-dose IL-2 versus IL-2 alone in unresectable stage III/IV melanoma (randomized phase III, *n* = 185).	Earlier studies showed circulating gp100-specific CD8^+^ T cells in most vaccinated patients; phase III immunomonitoring was limited but confirmatory.	gp100 increased ORR from 6% to 16% and mPFS from 1.6 to 2.2 months, with a trend toward longer OS; toxicity was similar between arms and driven by IL-2.	A single optimized shared antigen can augment cytokine therapy, but absolute efficacy remained modest.
NY-ESO-1 79–108 long peptide (78)	Long synthetic NY-ESO-1 peptide + CpG-B in Montanide, ± low-dose IL-2, in resected stage III/IV melanoma (phase I; 18 evaluable).	Integrated NY-ESO-1-specific CD8^+^ and CD4^+^ T-cell plus antibody responses in 18/18, with polyfunctionality, epitope spreading, and persistence for ≥ 1 year.	Clinical benefit consisted mainly of prolonged no evidence of disease (NED) or stable disease (SD), with only occasional PR.	The long-peptide format is a strong priming strategy but, as monotherapy, mainly produced durable immune activation and disease stabilization rather than high ORRs.
Four-peptide vs. twelve-peptide multiepitope vaccines (Mel39) (80)	Adjuvant 4- versus 12-peptide shared-antigen vaccines for resected high-risk melanoma (stage IIB-IV; *n* = 51).	Both formulations were highly immunogenic; broader peripheral immune responses were seen with 12 peptides.	Post-hoc 20-year analysis showed OS of about 65% at 10 years and 49% at 20 years overall; HR for OS favored 12 peptides but was not significant, and RFS did not differ significantly.	Increasing from 4 to 12 epitopes broadened induced CD8 + responses but did not significantly improve long-term survival.
Ten-peptide moDC vaccine (82)	Cocktail-matured moDC loaded with 10 defined class I/II melanoma peptides, with DC-licensing strategies, in stage III/IV cutaneous melanoma (phase I/II; *n* = 62).	Multi-epitope T-cell responses occurred in nearly all patients; most responses were induced de novo, and memory persisted for years.	With ≥ 12-year follow-up, about 19% of patients with non-resectable metastatic melanoma remained alive, but no WHO-defined CR/PR occurred.	This large mixed class I/II peptide pool was highly immunogenic and associated with long-term survival in a subset, but peptide breadth did not correlate with OS, suggesting diminishing returns from simply adding more shared peptides.

#### Combination strategies of DCV with ICIs and adoptive t-cell transfer

Combination strategies integrate DCV with ICI or adoptive T-cell transfer to overcome tumor-induced immunosuppression and enhance the persistence and function of vaccine-primed T cells. In preclinical melanoma models, combining DCV with PD-1-targeting antibodies increases the density and function of tumor-infiltrating effector T cells, reduces regulatory T-cell and myeloid-derived suppressor-cell populations, and improves tumor control and survival compared with either monotherapy [69, 70].

Adoptive T-cell transfer (ATT) programs have incorporated DC-based vaccination before or after infusion of tumor-infiltrating lymphocytes (TIL) or other ex vivo expanded T-cell products. In stage IV melanoma, first clinical trials showed that adding a tumor-antigen-pulsed DC vaccine to TIL therapy was feasible and safe and enhanced antigen-specific T-cell responses, although ORRs did not clearly exceed those expected with TIL therapy alone [71, 72]. Other combinations, including DCV with metronomic cyclophosphamide, low-dose IL-2, or cyclo-oxygenase-2 inhibition, achieved disease control in about one third to one half of metastatic melanoma patients in single-arm phase II studies, but did not meaningfully improve OS [39, 73].

Overall, available data suggest that optimized DCVs are most effective as part of rational combination strategies with ICI or ATT, in which DCs provide antigen-specific priming while partner therapies relieve inhibitory pathways or increase effector-cell numbers. However, randomized trials are still needed to define the magnitude of clinical benefit in melanoma.

#### Tumor-infiltrating lymphocytes

ATT with TILs is a personalized immunotherapy that exploits the pre-existing, tumor-experienced T-cell repertoire of individual patients to mediate antitumor activity in solid tumors [74]. The standard procedure involves resection of a tumor lesion, ex vivo expansion of resident TILs under high-dose IL-2, and reinfusion after non-myeloablative lymphodepletion with short-course high-dose IL-2 support [74, 75]. TIL products are typically polyclonal, enriched for CD8^+^ T cells, and contain clonally expanded populations recognizing multiple autologous tumor epitopes, including neoantigens, which likely underlies the depth and durability of clinical responses [74, 76]. Unlike monoclonal ICI, TIL therapy transfers a high-frequency tumor-reactive T-cell population into a host environment optimized for expansion.

The clinical development of TIL therapy was pioneered in metastatic melanoma, whose high mutational burden and frequent baseline T-cell infiltration made it an ideal proof-of-concept setting [75, 77]. Early single-center studies reported ORR of about 40–50% in heavily pretreated melanoma, including durable complete responses [75, 78, 79], establishing TIL transfer as one of the most active immunotherapies in melanoma before the checkpoint inhibitor era. These data supported the development of standardized TIL products and randomized comparisons with established ICI.

Lifileucel is a centrally manufactured, cryopreserved, autologous TIL product and currently the most advanced clinical implementation of TIL therapy in melanoma [80, 81]. In the phase II C-144-01 trial in unresectable or metastatic melanoma previously treated with PD-1 blockade and, if BRAF^V600^-mutated, targeted therapy, a single lifileucel infusion after standard lymphodepletion and high-dose IL-2 achieved an ORR of about 31–36%, with complete responses in about 5–7% and disease control in roughly 70–80% of patients [80, 82]. These results led to accelerated approval of lifileucel (AMTAGVI™) by the United States Food and Drug Administration (FDA) in 2024 for adults with unresectable or metastatic melanoma previously treated with a PD-1 inhibitor and, if BRAF^V600^-mutated, an appropriate BRAF-targeted regimen, making it the first approved TIL therapy for a solid tumor [81].

Randomized evidence further established the clinical relevance of TIL therapy. In the phase III trial by Rohaan et al., patients with advanced melanoma progressing after anti-PD-1 therapy were randomized to TIL therapy or ipilimumab; PFS was significantly longer and ORR substantially higher with TIL therapy, supporting its role as an active option for checkpoint-refractory melanoma [83].

The toxicity profile of TIL therapy is driven mainly by lymphodepleting chemotherapy and high-dose IL-2 rather than antigen off-target effects of the infused cells [80, 84]. Lymphodepleting chemotherapy induces in nearly all patients grade 3–4 AE, predominantly hematologic toxicities, which were usually transient and manageable with standard supportive care [82–84]. High-dose IL-2 further contributes to capillary leak syndrome, hypotension, tachycardia, renal and hepatic dysfunction, and the need for intensive monitoring, although these events are generally reversible and limited to the acute treatment period [78, 84, 85]. Treatment-related mortality across modern TIL studies, including lifileucel, has remained in a low range [82, 83]. Importantly, severe immune-mediated toxicities characteristic of checkpoint inhibitors, appear less frequent with TIL therapy [83, 84, 86]. Thus, although TIL therapy is intensive and should be delivered in specialized centers, its safety profile is predictable and acceptable given its potential for durable disease control.

### Optimization strategies for TIL therapy

#### Optimization of TIL-product composition and manufacturing

**Fig. 2:**
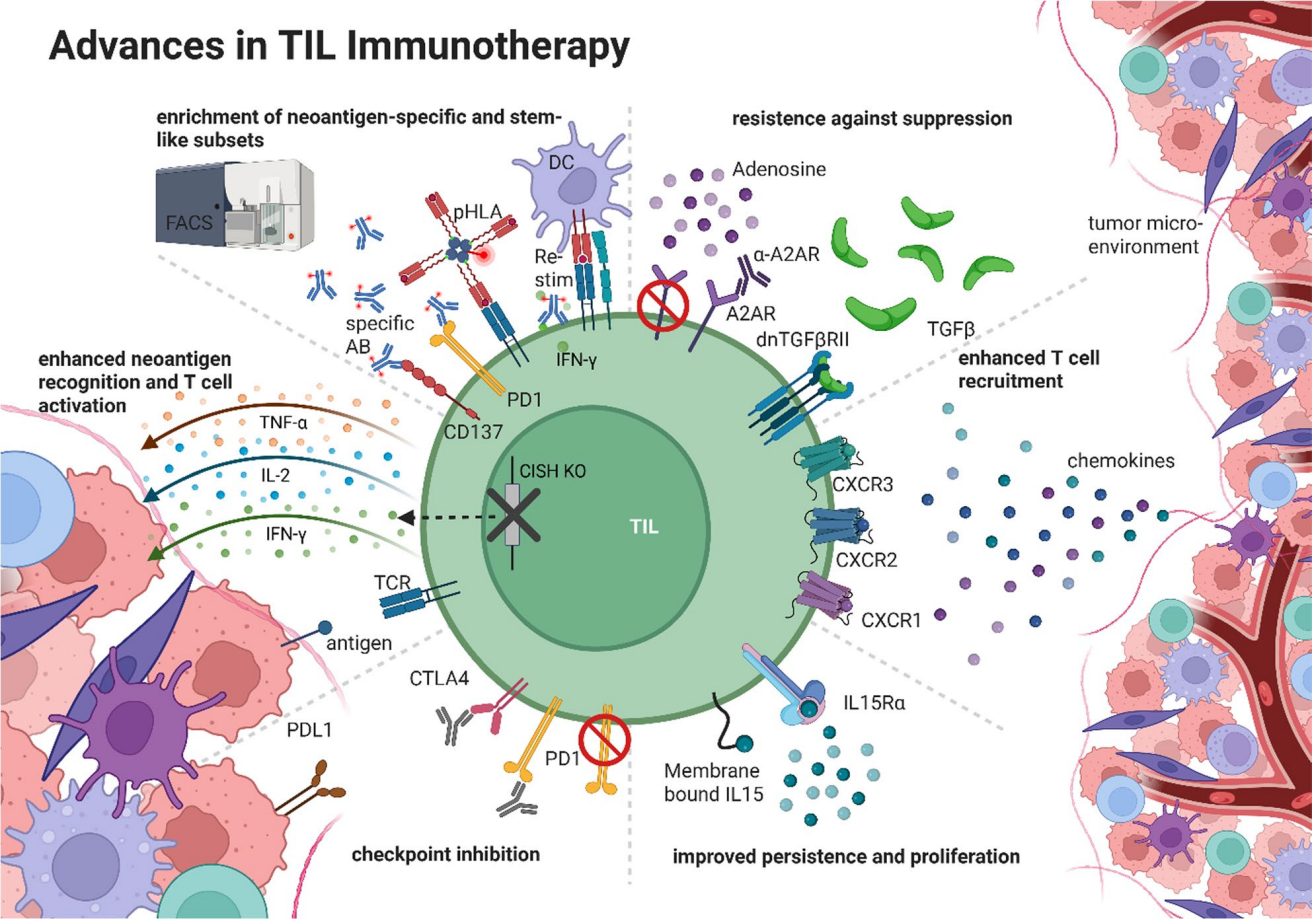
Advances in TIL immunotherapy through optimization of product composition, functional fitness, and tumor microenvironment resistance. Multiple strategies aim to improve TIL therapy in melanoma and other solid tumors by enhancing response durability and simplifying manufacturing. Tumor-reactive and neoantigen-specific TILs can be enriched using cytokine capture assays, activation-induced markers, PD-1, CD39, CD103, CD137, or peptide-HLA multimers. Long-term persistence is promoted by preserving stem-like TIL states, shortening expansion, and using IL-7/IL-15 instead of exclusive high-dose IL-2, with optional IL-21 and 4-1BB co-stimulation. Efficacy may be further enhanced by combining TILs with PD-1 blockade and by engineering resistance to tumor-mediated suppression through approaches such as CISH knockout, TGF-β or adenosine pathway targeting, chemokine receptor retargeting, and membrane-bound IL-15 expression

Several strategies aim to optimize TIL therapy in melanoma and other solid tumors by improving response rates and durability while simplifying logistics and reducing toxicity (Fig. 2). A central focus is TIL composition, especially enrichment of neoantigen-specific or stem-like T-cell subsets and avoidance of terminally exhausted or regulatory populations, which correlates with superior clinical responses in retrospective analyses [76, 87]. Antigen-specific enrichment can be achieved by cytokine capture, in which activated T cells are isolated by magnetic-activated cell sorting (MACS) or fluorescence-activated cell sorting (FACS) after retaining secreted cytokines on a surface-bound matrix [88]. Interferon-γ (IFN-γ) is the most widely used functional readout for tumor recognition in CD8⁺ and CD4⁺ T cells, with Good Manufacturing Practice (GMP)-grade and automated workflows reported [89]. However, cytokine production is confined to particular T-cell subsets, so enrichment based solely on IFN-γ or related readouts may fail to recover all antigen-specific populations [90].

Activation-induced marker capture is an alternative based on marker upregulation after brief co-culture with autologous tumor or personalized neoantigen pools presented by professional APCs [90, 91]. Antigen-agnostic enrichment using PD-1 can recover patient-specific tumor-reactive CD8⁺ T cells, but exhausted and bystander cells remain a limitation [92, 93]. CD39 and CD103 co-expression likewise identifies tissue-resident memory CD8⁺ T cells enriched for tumor reactivity, although these cells often display an exhausted phenotype that may impair persistence [94, 95]. Among available markers, CD137 (4-1BB) appears particularly selective, as depletion of CD137⁺ cells markedly reduces tumor reactivity within PD-1-, CD39-, or CD103-enriched TIL populations [91].

An alternative strategy uses fluorochrome-conjugated peptide-HLA (pHLA) multimers to identify antigen-specific T-cell receptor (TCR) specificities independent of functional readouts. Although highly specific for predicted neoantigens, this method is constrained by HLA restriction, imperfect epitope prediction, and substantial labor and cost [96, 97].

Stem-like phenotypes can be enriched by selecting CD39⁻ CD69⁻ CD8⁺ TILs, which mark a TCF1⁺ progenitor population associated with complete regression and long-term persistence after ATT [87]. Shortening pre-expansion preserves younger, less differentiated TILs [98, 99], whereas replacing exclusive high-dose IL-2 with IL-7 and IL-15, optionally with IL-21, and adding 4-1BB co-stimulation during outgrowth or rapid expansion can further improve CD8⁺ yield and fitness [100–102].

Overall, enrichment of neoantigen-specific and TCF1⁺/stem-like subsets while avoiding terminally exhausted or regulatory populations is a promising strategy to improve TIL durability. However, prospective evidence that such selection improves ORR over unselected bulk TIL products remains limited [76, 87].

#### Combination strategies with immune checkpoint inhibition

Combining TIL therapy with PD-1 inhibition is conceptually attractive because TIL infusion replenishes tumor-reactive lymphocytes, whereas PD-1 blockade may sustain their function and limit re-induction of hypofunctional or exhausted states in the tumor microenvironment. Consistent with the model proposed by Datsi and Garg, TIL plus PD-1 strategies aim to increase both the number and functional quality of tumor-specific T cells beyond a critical threshold, rather than merely releasing inhibitory brakes on an insufficient endogenous repertoire [24]. Early clinical data support this rationale: in the phase II IOV-COM-202 trial in previously untreated unresectable or metastatic melanoma, lifileucel plus pembrolizumab achieved an ORR of 65.2%, including a CR of 30.4%, with regression of target lesions in all evaluable patients and expected regimen-related toxicity [103]. These findings support biologically plausible synergy and justify randomized first-line trials comparing TIL therapy with combined ICI plus TIL approaches.

#### Genetic-engineering strategies

Genetic modification of TILs is a key strategy to improve their efficacy and durability. Cytokine-inducible SH2-containing protein (CISH) is an intracellular negative regulator of T-cell receptor (TCR)- and cytokine signaling, and preclinical models show that CISH knockout (CISH^KO^) enhances T-cell proliferation, cytokine production, metabolic fitness, and tumor recognition without broad non-specific autoreactivity [104, 105]. In the first-in-human phase I trial of CRISPR-Cas9-edited CISH^KO^ TILs in metastatic gastrointestinal epithelial cancers, large-scale manufacturing, engraftment, and an acceptable safety profile were demonstrated, without dose-limiting gene-editing-specific toxicities. Preliminary efficacy signals included stable disease in several patients and at least one durable complete response in microsatellite instability-high colorectal cancer, providing clinical proof-of-concept that intracellular checkpoint deletion can enhance TIL function in humans [106].

Additional engineering strategies aim to increase the therapeutic index of TIL therapy, including deletion of inhibitory receptors such as PD-1 [107], disruption of suppressive cytokine pathways such as TGF-β signaling [108], introduction of autocrine cytokine support circuits [109], and chemokine-receptor retargeting to improve homing [110]. A related approach, termed cytokine engineering, seeks to overcome dependence on systemic high-dose IL-2. For example, OBX-115 expresses membrane-bound human IL-15 under pharmacological control to provide cell-intrinsic survival and proliferation signals and potentially reduce the need for exogenous IL-2 [109, 111].

TIL therapy provides clinically meaningful durable responses in patients with advanced melanoma after failure of ICI and targeted therapies, with a predictable and manageable short-term toxicity profile [82]. Long-term lifileucel data, superiority over ipilimumab [83], and FDA approval as Amtagvi [81] have established TIL therapy as a key treatment for advanced melanoma. Ongoing engineered and combination approaches, including CISH^KO^ and other gene-edited products, may further improve efficacy, safety, and applicability across cancers. However, unlike CAR T cells, TIL production requires resectable tumor tissue.

#### CAR T cell therapy

CAR T cells combine an antibody-derived binding domain with intracellular activation and costimulatory modules such as CD28 or 4-1BB, enabling HLA-independent recognition of tumor-associated surface antigens and rapid T-cell activation [112]. Manufacturing involves isolation of autologous T cells from leukapheresis, ex vivo activation, gene transfer of the CAR transgene, usually by γ-retroviral or lentiviral vectors, and large-scale expansion under GMP conditions before lymphodepleting chemotherapy and reinfusion [113, 114]. Process variables such as cytokine composition and culture duration critically influence phenotype, function, and in vivo kinetics of the final product [115, 116].

Clinically, CAR T cells show high efficacy in hematologic malignancies such as diffuse large B-cell lymphoma, other B-cell lymphomas, and multiple myeloma, where pivotal or randomized trials have demonstrated superior disease control compared with standard of care [117, 118]. The principal toxicities are cytokine release syndrome (CRS) and immune effector cell-associated neurotoxicity (ICANS), which result from on-target immune activation and range from mild to life-threatening [119]. Rare post-therapy T-cell neoplasms have also been reported, leading to increased pharmacovigilance and label warnings in 2024, although the absolute risk appears low relative to clinical benefit [120]. To date, no CAR T-cell therapy has been approved for melanoma or other solid tumors, and all CAR-based strategies in melanoma remain investigational.

#### Melanoma Targets for designing the CARs

Despite successes in hematologic malignancies, translating CAR T-cell therapy to melanoma remains difficult because of antigen heterogeneity, shared expression with normal melanocytic and neural tissues, and a profoundly immunosuppressive tumor microenvironment (TIME) [121]. The following section therefore focuses on melanoma-relevant target antigens that form the basis for current and emerging CAR T-cell strategies in this disease (Table 2).

**Table 2:** Expression of Melanoma Targets

Target	Alias / Synonyms	Expression in Melanoma	Potential Off-Tumor Risk (examples)	Evidence Level	Clinical Status / Activity	Example Studies / IDs
GD2	Disialoganglioside GD2	Frequent/variable in cutaneous & metastatic melanoma	Peripheral nervous system, pain fibers; occasional skin/mesenchymal expression	Early clinical	Phase I: safe/feasible; biological activity, limited clinical efficacy	JITC 2024
B7-H3	CD276	Broad across solid tumors incl. melanoma; low in normal tissues	Low basal expression in healthy tissues (widely distributed, mostly weak)	Recruiting	Dose-finding (safety/MTD)	NCT04897321
IL13Rα2	IL-13 receptor α2	Subsets of melanoma (heterogeneous); elevated in some brain mets	Limited normal expression (e.g., testis, smooth muscle, variable)	Recruiting	Phase I (safety/dose)	NCT04119024
CSPG4	MCSP, HMW-MAA	High and frequent on melanoma cells	Pericytes, melanocyte precursors; limited normal expression	Strong preclinical	CAR T active preclinically	DOI:10.3390/cancers11081198 (131)
TYRP1	gp75	Melanosomal with surface pools shown (cutaneous, acral, uveal models)	Pigmented tissues (melanocytes); vitiligo-like effects possible	Strong preclinical	Efficacy in PDX/models; clinical pending	DOI: 10.1038/s41467-024-45221-2 (135)

B7 homolog 3 (B7-H3) is broadly expressed across solid tumors, including melanoma, and on tumor vasculature and stroma, whereas normal tissues show mostly low protein expression, supporting a potential therapeutic window [122, 123]. In the first-in-human STRIvE-02 phase I trial of systemic B7-H3 CAR Ts in relapsed or refractory solid tumors, 1 of 9 treated patients achieved a partial response, and toxicity was mainly limited to low-grade CRS and transient transaminitis, without clear acute organ injury attributable to B7-H3 expression in healthy tissues [124]. Several additional B7-H3 CAR T studies allow enrollment of B7-H3 positive melanoma, yet no melanoma specific safety or response data have been reported [125, 126]. Preclinical data further suggest that epigenetic modulators may pharmacologically increase B7-H3 density, although this has not been tested in melanoma and could increase on-target, off-tumor risk [127].

Chondroitin sulfate proteoglycan 4 (CSPG4), also referred to as NG2, is frequently expressed on primary and metastatic melanoma and other aggressive tumors, whereas normal expression is largely restricted to pericytes and selected stromal subsets, implying possible vascular off-tumor toxicity [128–130]. CSPG4-directed CAR-engineered lymphocytes show potent melanoma killing in preclinical models, and GMP-compliant transient CSPG4 CAR T production has been established. However, the dedicated melanoma trial is still recruiting, and no human efficacy or safety data are yet available [131, 132].

Interleukin-13 receptor alpha 2 (IL13Rα2) is overexpressed in glioblastoma and subsets of melanoma and other solid tumors, with minimal to absent protein in most normal tissues [133, 134]. In a phase I trial of locoregional IL13Rα2 CAR Ts in recurrent high-grade glioma, treatment was feasible, toxicities were mostly low grade, and objective responses, including durable regressions, occurred without a consistent off-tumor injury signal [134] An ongoing phase I trial is evaluating IL13Rα2 CAR Ts in stage IIIC/IV melanoma and other IL13Rα2-positive solid tumors, but melanoma-specific efficacy data have not yet been reported [133].

Tyrosinase-related protein 1 (TYRP1) is a melanocytic differentiation antigen involved in melanin synthesis and is expressed in cutaneous melanocytes and retinal pigment epithelium as well as multiple melanoma subtypes [135]. High-affinity TYRP1 CAR Ts show selective recognition of TYRP1-high melanoma cells and robust antitumor activity in preclinical models, apparently driven by antigen-density-dependent activation, but no clinical data are available and toxicity to skin or ocular melanocytes remains theoretical [135–137].

Disialoganglioside (GD2) is highly expressed on neuroblastoma, other neuroectodermal tumors, and a substantial subset of metastatic melanoma, whereas normal expression is largely restricted to central nervous system (CNS) neurons, peripheral nerves, and melanocytes [138]. Clinical experience in neuroblastoma and diffuse midline glioma shows that GD2 CAR T cells can induce meaningful responses with manageable toxicities under close CNS monitoring [139–141]. In the first phase I trial of third-generation GD2 CAR Ts in adults with GD2-positive metastatic melanoma and other solid cancers, manufacture was feasible and no dose-limiting toxicities or severe unexpected neuropathic events occurred, but clinical responses were limited, suggesting that melanoma efficacy will likely require combination strategies or next-generation constructs [142].

Taken together, B7-H3, CSPG4, IL13Rα2, TYRP1, and GD2 are biologically plausible but imperfect melanoma targets. Their therapeutic windows are constrained by heterogeneous antigen density, residual expression in normal tissues, and resistance mechanisms within the TIME, highlighting the need for next-generation CAR T-cell designs that improve both safety and efficacy.

#### CAR T Upgrades

**Fig. 3:**
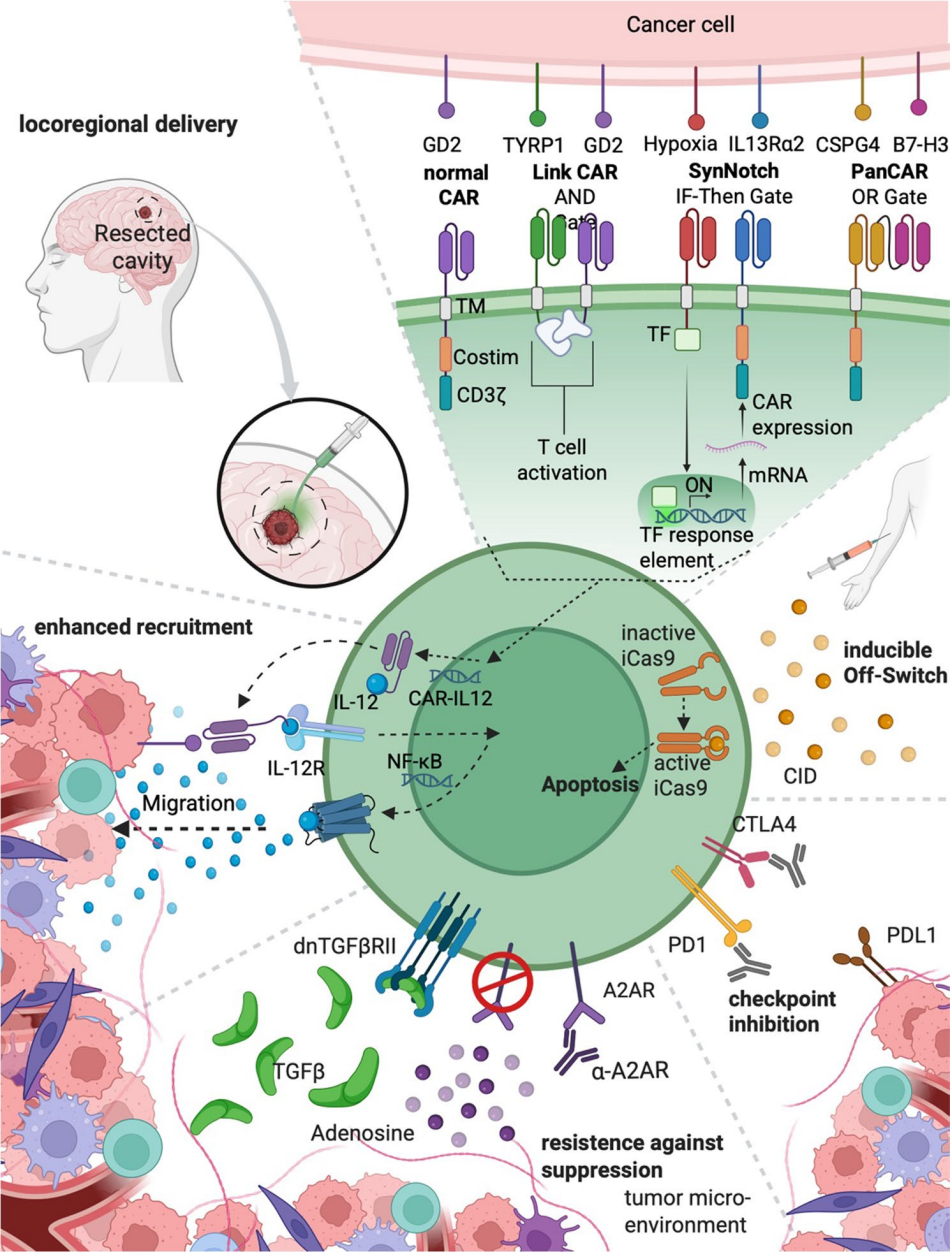
Modular next-generation CAR T-cell engineering strategies to overcome key barriers in melanoma. First-generation CAR T cells show limited activity in melanoma due to heterogeneous and plastic antigen expression, shared targets on normal melanocytes or neural tissues, a suppressive and metabolically hostile tumor immune microenvironment, and inefficient trafficking into metastatic lesions. Next-generation designs combine improved on-tumor recognition with tighter spatial control and enhanced safety. Multi-antigen targeting broadens coverage and limits antigen escape using dual or pan-CAR concepts that recognize more than one melanoma-relevant antigen. AND-gate CAR architectures restrict activity by requiring two inputs for full function, thereby reducing off-tumor activation. Logic-gated circuits also include synNotch IF-THEN programs that induce CAR expression only after a priming signal and OR-gates that maintain activity across heterogeneous target expression. Tumor localization can be increased by locoregional delivery and by trafficking enhancement through engineered chemokine receptors matched to tumor chemokine gradients. In the CAR–IL-12 concept shown, CAR–IL-12 is expressed after an upstream activation, recognizes the tumor antigen via its CAR-domain and simultaneously engages the T-cell IL-12 receptor via its IL-12 domain, driving downstream signaling that induces chemokine receptor expression and thereby improves migration into the tumor milieu. Resistance to suppression can be strengthened through disruption or decoying of inhibitory axes including TGF-β signaling via dominant-negative TGFβRII and adenosine signaling via A2A receptor targeting. Safety is reinforced through clinically controllable fail-safes such as inducible caspase-9 suicide switches activated by a small molecule CID (chemical inducer of dimerization) enabling rapid pharmacologic ablation of infused cells if severe toxicity occurs

The limited efficacy of first-generation CAR T-cell therapies in melanoma reflects persistent barriers including heterogeneous and plastic antigen expression, shared targets on normal melanocytic and neural tissues, a profoundly immunosuppressive TIME, and insufficient trafficking into metastatic lesions [143–145]. Next-generation CAR T designs therefore aim to broaden on-tumor recognition, improve localization, enhance resistance to immune suppression, and increase safety (Fig. 3).

A first area of optimization is antigen recognition. Dual, tandem, and conditional CAR constructs targeting more than one antigen can reduce antigen escape and improve control of heterogeneous tumors in preclinical models without introducing dominant new safety signals when targets are carefully selected [146, 147]. Supporting this concept, a pan-CAR T approach co-targeting B7-H3 and CSPG4 achieved broad antigen coverage and effective tumor control in heterogeneous triple-negative breast cancer models, suggesting relevance for melanoma, although clinical validation is still lacking [148].

Logic-gated CAR T cells, including synthetic notch (synNotch)-based and AND/NOT-gate architectures, further refine specificity by requiring defined antigen combinations before activation. In murine models, these systems reduce off-tumor toxicity while preserving antitumor activity, which is particularly relevant for melanoma targets such as TYRP1 and GD2 that are shared with essential normal tissues [146, 149, 150].

A second area is enhancement of CAR T-cell fitness within the TIME. Armored CAR T cells can conditionally deliver immunomodulators at the tumor site and thereby preserve proliferation, persistence, and cytotoxicity under chronically inflamed conditions [151, 152]. Constructs incorporating inducible IL-12, IL-18, IL-15, or αPD-L1/IL-12 fusion proteins have shown superior expansion, IFN-γ production, and durable tumor rejection in solid tumor models while limiting systemic toxicity when expression remains tightly linked to antigen engagement [153–156]. Early clinical experience, such as huCART19 IL-18 CARs in refractory lymphoma, supports the translatability of armored approaches [157]. In parallel, genetic disruption of inhibitory pathways can reduce susceptibility to soluble suppressive mediators. CRISPR-mediated deletion of the adenosine A2A receptor (A2AR) improved cytokine production and tumor control in Human epidermal growth factor receptor 2 (HER2)^+^ and mesothelin^+^ solid tumor models [158–160], whereas co-expression of dominant-negative transforming growth factor-β receptor type II (dnTGFβRII) or transforming growth factor-β traps improved proliferation and cytotoxicity under high TGF-β conditions [161, 162].

A third area is trafficking. Engineering CAR T cells to express chemokine receptors such as C-X-C chemokine receptor (CXCR) type 2 or C-C chemokine receptor (CCR) type 2, matched to melanoma-associated chemokine patterns, increased intratumoral accumulation and tumor control in xenograft models, although off-tumor chemokine gradients remain a concern [163–166]. Microenvironment-responsive designs that couple CAR signaling to hypoxia or P-selectin can further focus activity to tumor sites and reduce systemic exposure in murine models [167–169]. Locoregional administration, already explored for IL13Rα2 and B7-H3 CAR T cells in brain tumors, may also be relevant for melanoma with dominant CNS involvement, although dedicated melanoma trials are lacking [134, 170].

A fourth area is safety. Inducible suicide switches such as inducible caspase-9 (iCas9) allow rapid elimination of infused cells and have shown proof of principle in preclinical and early clinical studies [171–173]. Additional synthetic-biology approaches, including tunable ON switches, split CAR designs, and small-molecule-responsive domains, may allow in vivo control of CAR T-cell activity with improved safety [174, 175]. Such safeguards are especially important for targets such as GD2 and CSPG4, where low-level expression in normal tissues raises concern for neurotoxicity or skin toxicity. This concept is already reflected in melanoma-directed platforms: a B7-H3-targeted CAR T-cell strategy for metastatic uveal melanoma incorporates iCas9, whereas CSPG4-specific melanoma CAR T cells are being developed with transient mRNA electroporation to limit CAR expression duration [131, 176]. Fourth-generation platforms such as 4SCAR, combining a conventional CAR backbone with iCas9 and additional co-stimulatory modules, have entered early-phase solid-tumor trials with encouraging preliminary safety and antitumor activity [177–179].

Collectively, these upgrade strategies provide a modular toolkit to address the central obstacles identified for melanoma CAR T-cell therapy:

(I) suboptimal target specificity.
(II) antigen heterogeneity,
(III) a suppressive microenvironment,
(IV) poor trafficking.
(V) safety concerns.

The available data suggest that melanoma targets are unlikely to succeed as simple single-antigen, first-generation CARs, but may become more effective when integrated into logic-gated, armored, trafficking-enhanced, and safety-switch-equipped platforms (Table 3). The critical next step is rational clinical evaluation of such composite designs in biomarker-selected melanoma populations, with careful pharmacovigilance and systematic correlation of antigen expression, CAR T-cell dynamics, and clinical outcomes.

**Table 3:** Advantages and limitations of the therapeutic options for melanoma treatment

Therapy modality	Key advantages (pros)	Key disadvantages / limitations (cons)
Immune checkpoint inhibitors (ICI) (PD-1 and CTLA-4–based)	Can induce meaningful and sometimes durable responses by rescuing pre-existing antitumor T cells.	Primary and acquired resistance are common; efficacy is limited in cold or myeloid-inflamed TIME, and intensified regimens may be poorly tolerated.
Targeted therapy	Provides rapid disease control in appropriate molecular subsets and can be integrated into sequencing strategies.	Durable benefit is often limited in therapy-resistant disease, and repeated treatment lines add cumulative toxicity.
Dendritic cell (DC) vaccines	Excellent safety profile; can prime broad antigen-specific immunity and support T-cell, B-cell, and NK-cell responses with memory potential.	Objective responses as monotherapy are modest in advanced melanoma; best viewed as priming or backbone partners for combinations and dependent on a permissive TIME.
Tumor-infiltrating lymphocyte (TIL) therapy	Replaces the missing tumor-reactive repertoire; recognizes diverse targets including neoantigens; achieves ~ 30–40% ORR in checkpoint-refractory melanoma with durable complete responses in a subset.	Requires tumor resection, complex manufacturing, lymphodepletion, and high-dose IL-2; intensive treatment best delivered in specialized centers.
TCR-engineered T cells (TCR-T)	Can target intracellular antigens and neoantigens presented by HLA and may support long-lived memory when manufactured in a stem-like state.	HLA-restricted and usually narrow in specificity, with risk of immune escape; safety depends on strict control of off-target and cross-reactive toxicity.
CAR-T / CAR-NK cells	Not HLA-restricted, do not usually require patient-specific tumor material for target discovery, and allow programmable, potentially off-the-shelf development.	In melanoma and other solid tumors, efficacy is limited by scarce tumor-selective surface antigens, antigen heterogeneity, CRS/neurotoxicity, and limited persistence in TIME.
NK-cell–based immunotherapy (including adoptive NK, CAR-NK, engagers; exploratory)	HLA-independent, potentially off-the-shelf, often with a more favorable toxicity profile; may target antigen-loss and HLA-deficient clones and cooperate with DCs and T cells.	Still exploratory; limited by poor in vivo persistence, incomplete trafficking, suppressive TME factors, and the need for better targets, stronger platforms, and larger controlled studies.

#### Engineered T-cell receptor T-cell therapy in melanoma

Engineered TCR T-cell therapy redirects autologous T cells to tumor cells by introducing a defined, high-affinity TCR that recognizes a peptide-HLA complex derived from a tumor antigen. Unlike CAR T cells, which bind intact surface proteins independently of HLA, TCR-engineered T cells recognize intracellular proteins presented on HLA class I and, in some designs, class II, thereby expanding the target space to cancer-testis antigens, lineage antigens, and neoantigens inaccessible to conventional antibodies or CARs [180].

Manufacturing broadly parallels CAR T-cell production: T cells are collected by leukapheresis, activated ex vivo, transduced with a γ-retroviral or lentiviral vector encoding the tumor-specific TCR, expanded under controlled culture conditions, and infused after non-myeloablative lymphodepletion to promote engraftment and in vivo expansion [180, 181].

A key distinction is the emphasis on preserving an early memory, stem-like phenotype, which is associated with superior engraftment, self-renewal, and long-term persistence [180, 182]. Clinical studies of NY-ESO-1- and other tumor-specific TCR T cells have shown multi-year persistence and durable antitumor responses, consistent with formation of a functional memory pool [183]. In contrast, conventional CAR T-cell products are often skewed toward more differentiated effector phenotypes and are more prone to exhaustion, which can limit long-term persistence. Accordingly, process design for TCR T cells must balance efficient viral transduction with maintenance of a less differentiated, memory-like state [181, 184, 185].

#### Clinical activity and safety in melanoma and related solid tumors

Early proof-of-concept for TCR-engineered T cells in melanoma came from a landmark trial using an HLA-A*02:01-restricted NY-ESO-1-specific TCR. In patients with metastatic melanoma or synovial sarcoma refractory to standard therapy, NY-ESO-1 TCR-transduced T cells plus high-dose IL-2 induced objective responses in 5 of 11 melanoma patients and 4 of 6 synovial sarcoma patients, including durable complete responses in melanoma, establishing that TCR-engineered T cells can mediate deep and lasting regressions when a suitable target antigen is present [186].

More recently, MART-1-specific TCR-engineered peripheral-blood T cells were evaluated in metastatic cutaneous and uveal melanoma. Although the study achieved an ORR of 18%, it was terminated early because of dose-dependent, on-target/off-tumor toxicity affecting skin, eyes, and ears, including one treatment-related death at the highest dose level, illustrating the narrow therapeutic window of differentiation antigens shared with normal melanocytes [187].

Preferentially Expressed Antigen in Melanoma (PRAME)-directed TCR-T therapy is currently among the most advanced TCR-T programs in melanoma. PRAME is broadly expressed across melanoma subtypes and other solid tumors while being largely absent from healthy adult tissues [188, 189]. In the phase I ACTengine IMA203 trial, an autologous PRAME-specific TCR product achieved as presented at the 2025 ASCO Annual Meeting an ORR of 54% in heavily pretreated melanoma at the recommended phase II dose, with tumor shrinkage in most patients, a median duration of response of about 12 months, activity across cutaneous, mucosal, and uveal melanoma, low rates of high-grade CRS, and no consistent signal of off-target organ toxicity [190]. These findings prompted the randomized phase III SUPRAME trial comparing IMA203 with investigator’s choice in previously treated cutaneous melanoma [191].

Overall, current data support TCR-engineered T cells as a clinically active modality in melanoma when suitable antigens such as NY-ESO-1 or PRAME are targeted. Safety reflects both regimen-related toxicity from lymphodepletion and, where used, IL-2, and antigen-dependent risks. Cytopenias, infections, and transient systemic symptoms are common but usually manageable [182]. A distinct risk is unexpected cross-reactivity with structurally related self-peptides: affinity-enhanced MAGE-A3-specific TCRs caused fatal cardiac toxicity through off-target recognition of a titin-derived peptide, and other MAGE- and MART-1-directed products have produced severe neuro- and skin toxicities in melanoma [187, 192–194]. These events have driven increasingly stringent preclinical specificity testing and the development of additional genetic safety strategies.

#### Genetic-engineering strategies

**Fig. 4:**
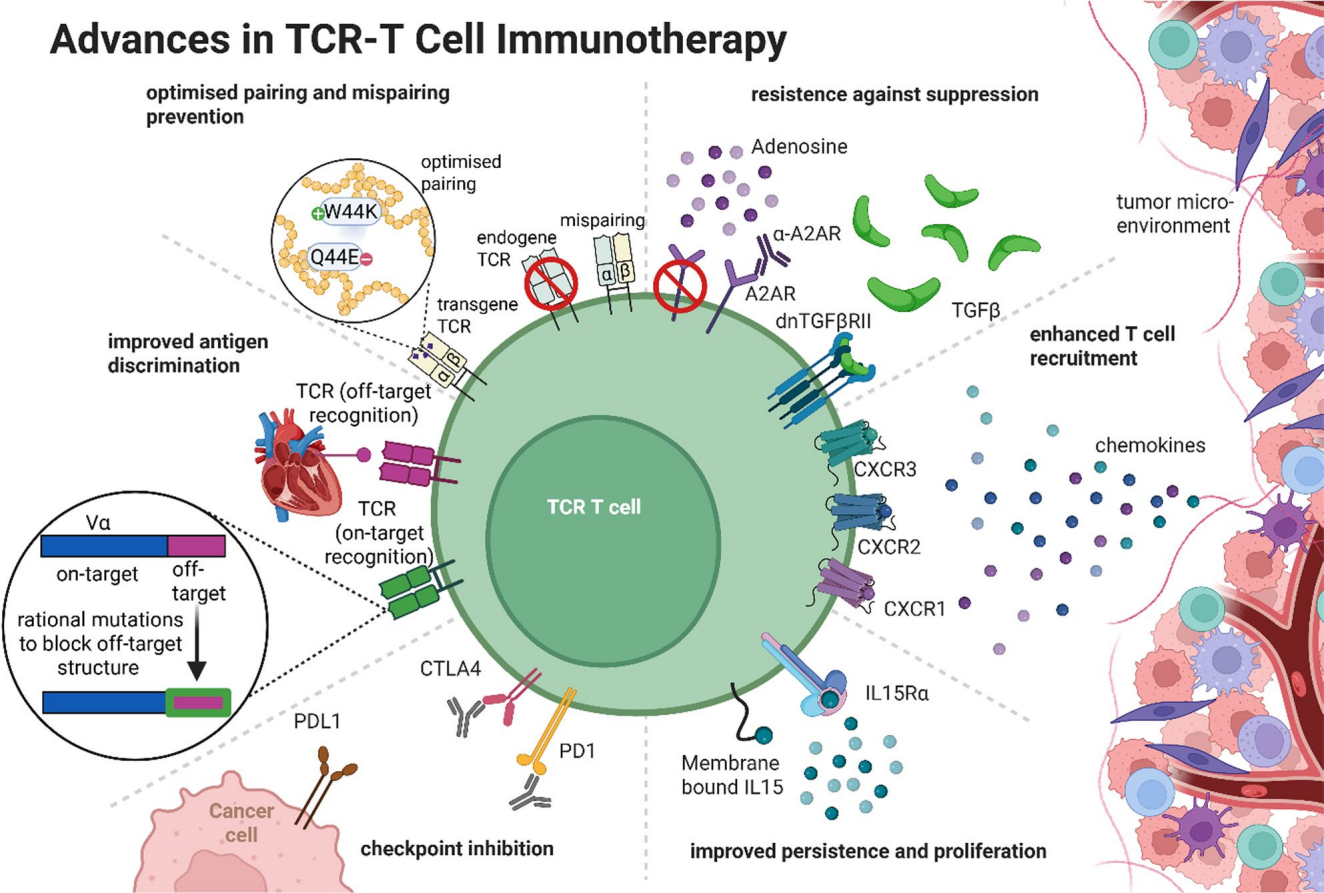
Advances in TCR-engineered T-cell immunotherapy for melanoma and other solid tumors. Engineered TCR T-cell therapy redirects autologous T cells by introducing a defined, tumor-reactive TCR that recognizes peptide–HLA complexes, thereby enabling targeting of intracellular antigens such as cancer-testis antigens, lineage antigens, and neoantigens that are not accessible to CAR T cells. Next-generation design features include structure-guided optimization to improve antigen discrimination and reduce off-target recognition, optimized TCR α/β pairing to favor correct chain assembly and limit mispairing and prevention of endogenous/transgenic TCR mispairing through silencing or deletion of native TCRα/β chains to generate monospecific products. Additional engineering modules aim to enhance function within the tumor microenvironment, including checkpoint pathway modulation (PD-1, CTLA-4), resistance to soluble suppression via adenosine (A2AR) and TGF-β (dnTGFβRII) axis interference, improved trafficking through chemokine receptor retargeting (e.g., CXCR1/2/3), and improved persistence through cytokine support circuits such as membrane-bound IL-15 and IL-15Rα

Many engineering concepts described for CAR T cells and genetically modified TILs, including checkpoint disruption, cytokine armoring, chemokine-receptor retargeting, and safety switches, also apply to TCR-engineered T cells (Fig. 4).

Central to TCR-T therapy is the engineered receptor itself. Current clinical programs mainly target cancer-testis antigens (NY-ESO-1, MAGE-A3/A4, PRAME), lineage-restricted differentiation antigens (MART-1, gp100), and self-proteins that are expressed at low levels in some normal tissues but upregulated in multiple tumors, such as GD2 and CSPG4 [180]. Compared with CARs, TCRs naturally recognize peptide-HLA complexes at very low antigen density, but affinity enhancement by mutagenesis can improve tumor recognition at the cost of increased cross-reactivity. The MAGE-A3/titin experience showed that even modest affinity enhancement can cause severe organ toxicity, underscoring the narrow therapeutic window of some targets [192, 193]. Current strategies therefore balance affinity optimization with extensive in vitro specificity testing and, where possible, structure-guided design to improve discrimination between target and off-target peptide-HLA complexes [189, 193].

Unlike CAR T cells, TCR-T products retain the native TCR repertoire in addition to the introduced tumor-specific TCR, creating a risk of mispairing between endogenous and transgenic α and β chains. This can reduce expression of the intended receptor and generate mixed TCRs with unpredictable specificities. One mitigation strategy is pairing optimization of the transgenic TCR to favor correct α/β assembly. In the PRAME-directed product IMA203, two charge-complementary amino acid substitutions at the α/β interface were introduced to promote preferential pairing and improve receptor stability and binding properties [189]. More stringent approaches combine TCR insertion with silencing or deletion of endogenous TCRα/β chains using shRNA, zinc-finger nucleases, or CRISPR-Cas9 to generate monospecific T cells expressing only the desired transgenic TCR [195, 196].

#### Positioning of engineered TCR-Ts within melanoma cell therapy

Within melanoma cell therapy, engineered TCR-T cells occupy an intermediate position between CAR T cells and TIL therapy. Like CAR T cells, they are generated from peripheral blood and allow modular genetic engineering [197]. Like TILs, they are HLA-restricted and rely on physiological TCR signaling, but they can target intracellular antigens inaccessible to CARs [180]. In melanoma, TCR-T has shown deep and sometimes durable responses, particularly against cancer-testis antigens such as NY-ESO-1 [198] and PRAME [189], while also illustrating that antigen selection and receptor design are central to safety.

As PRAME-targeted TCR-T programs progress into phase III trials [191] and multiplex-edited, armored, and safety-switch-equipped products emerge, TCR-T is likely to become an important complement to DC vaccines, CAR T cells, and TIL therapy in advanced melanoma. Its ultimate role will depend on whether ongoing trials translate response rates into durable survival benefit and reliably control off-target risks.

#### Natural killer cell-based therapy in melanoma

Natural killer (NK) cells are innate lymphocytes that detect and kill transformed or infected cells through a balance of activating and inhibitory receptors without prior antigen sensitization, enabling recognition of tumor cells with altered or absent HLA class I expression [199]. Their antitumoral activity is mediated through direct cytotoxicity, engagement of death receptors such as Fas and TNF-related apoptosis-inducing ligand (TRAIL), antibody-dependent cellular cytotoxicity via CD16, and secretion of IFN-γ and TNF that support DC activation and T-cell priming [200, 201].

In melanoma, NK cells recognize tumor targets through activating receptors such as natural killer group 2, member D (NKG2D), DNAX accessory molecule-1 (DNAM-1), and the natural cytotoxicity receptors NKp30, NKp44, and NKp46, which interact with induced self-ligands including HLA class I chain-related protein A (MICA), HLA class I chain-related protein B (MICB), UL16-binding protein (ULBP) family members, and the DNAM-1 ligands poliovirus receptor (PVR, also known as CD155) and Nectin-2 [202]. Human NK cells comprise cytotoxic CD56^dim^ CD16^+^ cells and more immunoregulatory CD56^bright^ CD16^−/low^ cells, with additional tissue-resident populations in skin and tumors [203]. In advanced melanoma, higher frequencies of CD56^bright^ NK cells are associated with reduced degranulation and shorter OS, whereas predominance of mature CD56^dim^ NK cells and high intratumoral NK cell density or NK gene signatures correlate with favorable prognosis and better responses to ICI [204, 205].

Melanoma evades NK surveillance through upregulation of HLA class I and inhibitory ligands, expression of HLA-E and HLA-G, and shedding or downregulation of NKG2D ligands, thereby reducing activating input and restoring dominant inhibition [206]. In addition, tumor-derived mediators such as TGFβ, PGE2, and indoleamine 2,3-dioxygenase (IDO) suppress NK activating receptors and promote poorly cytotoxic or regulatory NK subsets [206, 207]. The TIME further impairs NK function through hypoxia [208], nutrient deprivation [209], acidosis [210] and accumulation of MDSCs and T_regs_ [211], driving an exhausted phenotype with reduced cytotoxicity and cytokine production. Preclinical melanoma studies indicate that intact NK activity supports the efficacy of targeted therapy [212] and ICI [213, 214], whereas NK-cell loss or dysfunction promotes resistance, metastasis, and immune escape [214, 215], , providing a strong rationale for NK cell-based therapy in melanoma [200, 214].

#### Conventional and memory-like adoptive NK cell transfer

Adoptive NK cell therapy aims to restore or augment tumor-reactive NK cell function through ex vivo-expanded and activated autologous or allogeneic NK cell products [199]. Early trials with autologous NK cells in metastatic melanoma demonstrated feasibility and safety but only minimal clinical activity, likely because of dominant inhibition by self HLA ligands and limited in vivo persistence [199, 216]. This led to development of allogeneic NK strategies exploiting killer-cell immunoglobulin-like receptor (KIR) and HLA mismatch to reduce inhibitory signaling and enhance antitumor activity, an approach with proven benefit in acute myeloid leukemia that is now being translated to solid tumors including melanoma [216–219].

Allogeneic NK manufacture usually starts from leukapheresis products of healthy donors or umbilical cord blood (CB), followed by cytokine-based expansion with IL-2 or IL-15 and feeder-cell-based systems or artificial APCs to support large-scale expansion and a cytotoxic phenotype [220]. Process optimization has generated clinically relevant NK doses with preserved activating receptor expression, potent melanoma cell killing in vitro, and the option of cryopreservation for off-the-shelf use [221, 222]. In a phase I trial of haploidentical NK transfer in cancer patients including metastatic melanoma patients, treatment was feasible and generally well tolerated, but clinical activity remained limited: 4 of 10 melanoma patients achieved only transient stable disease and all subsequently progressed [218].

Cytokine-induced memory-like (CIML) NK cells represent a key refinement. Brief ex vivo stimulation with IL-12, IL-15, and IL-18 induces a memory-like state with enhanced IFN-γ production, cytotoxicity, and persistence upon restimulation [223, 224]. CIML NK cells can be generated from healthy donors and melanoma patients, retain cytotoxicity against autologous and allogeneic melanoma targets, and outperform conventional IL-2-activated NK cells in preclinical models [225]. Building on preclinical data demonstrating that cytokine-induced memory-like NK cells from healthy donors and melanoma patients efficiently control autologous and allogeneic melanoma targets in vitro and in vivo [225], early phase I trials of allogeneic CIML NK cells in other heavily pretreated solid tumors have shown acceptable safety, transient disease control, and occasional regressions [226]. However, robust melanoma-specific efficacy data are still lacking, and a dedicated phase I trial in advanced melanoma is ongoing (NCT05629546).

#### Engineered NK cells and CAR NK strategies in melanoma

**Fig. 5:**
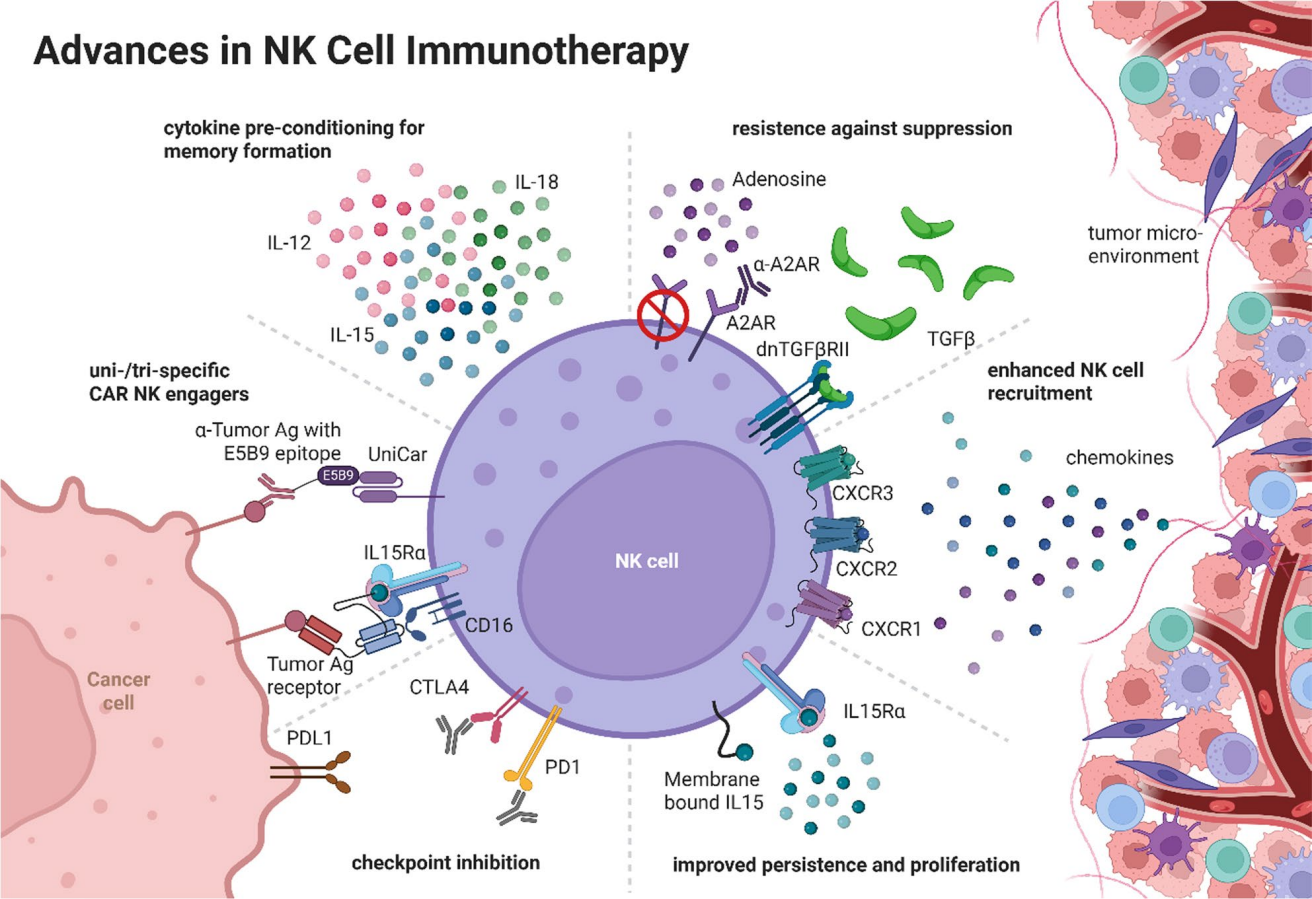
Advances in adoptive NK cell immunotherapy and engineered NK platforms for melanoma. Adoptive NK cell therapy uses ex vivo expanded and activated NK cells and is increasingly pursued with allogeneic products to mitigate self HLA mediated inhibition and limited persistence observed with early autologous approaches. Cytokine-induced memory-like (CIML) NK cells are generated by brief priming with IL-12, IL-15, and IL-18, imprinting enhanced IFN-γ production, increased cytotoxicity, and improved persistence upon restimulation. Genetic engineering further extends NK functionality through CAR expression and modular targeting formats, with the advantage of low graft-versus-host disease risk and generally favorable early safety signals across tumor types. In melanoma-relevant designs, tri-specific NK cell engagers link a tumor-associated antigen to CD16 to trigger NK activation while simultaneously delivering IL-15 as an intrinsic support signal for survival and expansion, and, in parallel, the UniCAR NK-92 platform uses a switchable adaptor module to redirect a universal CAR to different tumor antigens, decoupling the effector cell product from antigen specificity and adding a pharmacologically controllable safety layer. Additional engineering modules improve persistence through membrane-bound or secreted IL-15 and IL-15Rα support, enhance recruitment into inflamed lesions via chemokine receptor retargeting such as CXCR1, CXCR2, or CXCR3, and strengthen resistance to tumor microenvironment suppression by disrupting TGF-β signaling or targeting adenosine A2A receptor pathways. Combination strategies further aim to integrate NK therapies with checkpoint inhibition, targeted therapy, chemotherapy, or radiation to counteract tumor-induced suppression and exploit synergistic effects, although melanoma-specific clinical outcome data remain limited

Genetic engineering can further enhance NK-cell recognition and persistence by introducing CARs, cytokine support, or resistance to inhibitory pathways [227] (Fig. 5). Compared with CAR T cells, CAR NK products can be generated from allogeneic donors with low risk of Graft-versus-host disease, and early-phase trials across tumor types have shown favorable safety, with low rates of severe CRS or neurotoxicity and minimal long-term toxicity [228, 229]. Preclinical studies show that CAR NK cells targeting GD2 [230] or B7-H3 [231] mediate potent killing of melanoma cell lines and xenografts while retaining lower alloreactive risk than CAR T cells, supporting their development as off-the-shelf products.

NK-specific engineering strategies include membrane-bound or secreted IL-15 to support survival [232], expression of chemokine receptors such as CXCR1, CXCR2, or CXCR3 to improve trafficking to inflamed melanoma lesions [233–235] and disruption of TGF-β signaling [236, 237] or targeting of A2ARs to overcome microenvironmental suppression [238]. Early clinical trials of engineered NK cells outside melanoma already provide encouraging safety signals, including CD19 CAR NK cells in hematologic malignancies [239] and trispecific NK-cell engagers combining tumor targeting, CD16 engagement, and IL-15 delivery, which have shown objective responses with limited severe toxicity [240, 241]. These findings support cautious optimism for translation to melanoma.

Melanoma-specific CAR NK programs remain early but are progressing. Preclinical and translational studies describe CAR NK constructs against GD2, CSPG4, MCSP, and other melanoma-associated antigens that achieve robust tumor control in xenograft models and can be produced from CB or induced pluripotent stem cell-derived NK cells at scale [242, 243]. A universal CAR NK concept has also been developed with the UniCAR NK-92 platform, in which NK-92 cells expressing a universal CAR are redirected to different antigens via soluble target modules, adding flexibility and pharmacologic safety control [230]. In parallel, supercharged NK cells generated through combined cytokine and co-stimulatory receptor signaling have shown strong melanoma control in humanized mice, suggesting an additional route to enhance NK potency with or without CARs [244, 245].

#### Combinatorial approaches

Combination strategies integrate NK cell-based therapies with immune checkpoint inhibition, targeted therapy, or radiation to counteract tumor-induced suppression and exploit synergy [246, 247]. Preclinical studies show that PD-1 and CTLA-4 blockade can enhance NK-cell activity by reshaping the TIME, and that tumor-infiltrating NK cells may themselves express checkpoint receptors such as PD-1 and T cell immunoreceptor with Ig and ITIM domains (TIGIT), providing a rationale for direct checkpoint blockade on NK cells [248]. In BRAF^V600E^ melanoma models, NK cells are required for sustained tumor control during BRAF inhibition, and loss of NK function accelerates relapse and metastatic outgrowth [212]. Clinically, early-phase trials are evaluating ex vivo-expanded or engineered NK-cell products with anti-PD-1 antibodies [249] or chemotherapy [250] in advanced solid tumors. In biliary tract cancer, allogeneic SMT-NK cells plus pembrolizumab achieved an overall response rate of 17.4% in the full-analysis set and 50.0% in the per-protocol population without severe combination-related AEs [249]. In locally advanced colon carcinoma, adjuvant NK-cell infusions added to chemotherapy improved 5-year PFS and OS without new safety signals [250]. Melanoma-specific clinical outcome data, however, remain limited.

#### Current limitations and future directions

Despite conceptual advantages, including HLA-independent recognition, off-the-shelf applicability, and a more favorable toxicity profile than many T-cell products [251–253], NK cell-based therapies for melanoma remain at an exploratory stage [216, 254]. Major obstacles include limited in vivo persistence and expansion, insufficient trafficking into metastatic sites, strong suppression by TGF-β, adenosine, and other microenvironmental factors, and the need to identify melanoma antigens with sufficient selectivity for engineered NK targeting [255, 256]. Systematic reviews in solid tumors conclude that current evidence, derived mainly from early-phase small trials, supports favorable safety and signs of clinical and biological activity, but also underscores the need for more potent and standardized NK platforms, rational combination strategies, and larger controlled studies to define their therapeutic potential [253, 255, 257].

## Conclusion

Despite major advances with ICI and targeted therapy, therapy-resistant melanoma remains a major clinical and socioeconomic challenge, as primary and acquired resistance to PD-1- and CTLA-4-based regimens occurs in a substantial proportion of patients [21, 26]. Many patients never respond, relapse after an initial response, or cannot tolerate intensified checkpoint-based regimens and therefore receive multiple lines of systemic treatment with cumulative toxicity but limited durable benefit [20–22, 25]. These patients require strategies that not only modulate pre-existing immunity but rebuild and sustain a competent tumor-reactive immune compartment [31].

Resistance to ICI in melanoma is not explained by T-cell hypofunction alone, but reflects multiple T-cell-intrinsic, microenvironmental, and tumor-intrinsic mechanisms within the TIME. Chronic antigen exposure, metabolic stress, and inhibitory cytokine signaling promote exhausted or pre-exhausted T-cell states with reduced proliferative capacity and impaired cytokine production [24, 28]. Beyond this, resistance may result from insufficient numbers or quality of tumor-reactive lymphocytes, impaired antigen presentation, myeloid- and T_reg_-mediated immunosuppression, and tumor-intrinsic resistance programs [21, 24, 26, 28].

Cellular immunotherapies directly address this problem by supplying tumor-reactive lymphocytes or by priming them de novo, while also offering opportunities to remodel the TIME and re-establish immune memory [30, 31]. DC vaccines exemplify this approach by introducing new T-cell specificities through shared antigens, neoantigens, or whole tumor lysates and priming polyfunctional CD4⁺ and CD8⁺ T cells [33, 45]. Their major strengths are excellent safety, outpatient feasibility, and the potential to induce broad and durable immunity, including immune memory [33, 36, 37, 258]. However, objective response rates as monotherapy in advanced melanoma remain modest, so DC vaccines are increasingly viewed as priming or backbone components of combination regimens rather than stand-alone treatments in late-line disease [33, 35, 69, 70].

TIL therapy directly replaces the missing tumor-reactive repertoire. TIL products are enriched for clonotypes that have already infiltrated the tumor and can target a broad antigen spectrum, including patient-specific neoantigens, making them less vulnerable to single-target escape than engineered products [74, 76, 259]. Clinically, TIL therapy achieves response rates that exceed those of most salvage therapies in checkpoint-refractory melanoma, with lifileucel and related products showing objective response rates of about 30–40% and durable complete responses in a meaningful subset of patients [82, 83]. However, TIL therapy requires tumor resection, complex manufacturing, and lymphodepleting conditioning with high-dose IL-2, and is therefore best delivered in specialized centers to patients able to tolerate an intensive but time-limited regimen [74, 78, 86, 259].

Genetically engineered TCR-T cells and CAR T/CAR NK cells offer complementary advantages and limitations. TCR-T cells can target intracellular cancer-testis antigens and neoantigens presented by defined HLA molecules and, when manufactured in a stem-like state, may form long-lived memory populations [87, 180, 182, 184]. Their limitations are HLA restriction, narrow antigen specificity, and safety risks related to off-target or cross-reactive peptide recognition [180, 182, 192, 193]. CAR-based strategies overcome HLA restriction and usually do not require autologous tumor material for target discovery, enabling off-the-shelf development and rapid retargeting of both T and NK cells [112, 242, 243, 252]. In melanoma and other solid tumors, however, efficacy is constrained by the scarcity of truly tumor-selective surface antigens, antigen heterogeneity and plasticity, and acute toxicities for CAR Ts such as cytokine release syndrome and neurotoxicity [119, 155]. Many conventional CAR T products also acquire terminal effector differentiation and exhaustion, limiting persistence and memory formation in the TIME [145, 184].

NK-cell-based immunotherapy remains exploratory in melanoma. Its potential advantages include HLA-independent recognition, off-the-shelf manufacturability, and a comparatively favorable toxicity profile, but these are counterbalanced by limited in vivo persistence and expansion, incomplete trafficking into metastatic sites, strong suppression by TGF-β, adenosine, and other microenvironmental factors, and the need for sufficiently selective melanoma targets for engineered NK approaches [206, 214, 216, 229, 242, 255]. Current early-phase studies mainly support excellent safety and encouraging biological activity, but more potent and standardized NK platforms, optimized combinations, and larger controlled trials are needed before NK-cell therapies can claim a defined position alongside TIL, TCR-T, and CAR-based approaches in advanced melanoma [216, 242, 253].

Taken together, no single cellular therapy fully resolves the multifactorial problem of therapy-resistant melanoma. DC vaccines excel at safe, antigen-diverse priming and memory induction but depend on a minimally permissive TIME [33, 35, 258]. TIL therapy provides a broad tumor-experienced repertoire with preserved homing and broad target recognition, but at the cost of individualized and intensive treatment [74, 83, 259]. TCR-T and CAR T/CAR NK products offer potent and programmable effector cells, yet remain limited by antigen specificity, toxicity risks, and incomplete persistence [112, 155, 180, 182, 229, 242]. NK-cell-based strategies may complement these platforms by targeting antigen-loss and HLA-deficient clones and by cooperating with DCs and T cells, but they still require further optimization for persistence, trafficking, and resistance to the suppressive TME [206, 251–253] (Fig. 5).

The most plausible future direction is rational combination and sequencing of cellular platforms with checkpoint modulation and TIME-directed strategies. DC vaccines may be used to prime de novo tumor-specific T- and NK-cell responses before or after ATT, thereby enriching the effector pool that can then be expanded or sustained by TIL, TCR-T, or CAR-based approaches [33, 35, 71, 260]. In heavily pretreated, checkpoint-refractory melanoma, strategies such as TIL plus PD-1 blockade or DC vaccination followed by TIL infusion and delayed ICI are particularly attractive and warrant further clinical evaluation [72, 103, 259]. The therapeutic options are summarized in Table 3.

## Data Availability

No datasets were generated or analysed during the current study.

## Abbreviations

12MP+6MHP: 12-peptide vaccine plus melanoma-specific helper peptides
4-1BB: CD137
A2AR: Adenosine A2A Receptor
AE: Adverse Events
ATT: Adoptive T-cell transfer
B7-H3: B7 Homolog 3
BRAF: B-Raf proto-oncogene
CAR: Chimeric Antigen Receptor
CB: Cord Blood
CCR: Chemokine Receptor
CID: Chemical Inducer of Dimerization
CIML: Cytokine-induced memory-like
CISH: Cytokine-inducible SH2-containing protein
CISHKO: CISH knockout
CNS: Central Nervous System
CR: Complete Responses
CRS: Cytokine Release Syndrome
CSPG4: Chondroitin Sulfate Proteoglycan 4
CTL: Cytotoxic T cells
CTLA-4: Cytotoxic T-Lymphocyte-Associated Protein 4
CXCR: C-X-C Chemokine Receptor
DCV: Dendritic-cell-based Vaccination
DNAM-1: DNAX Accessory Molecule-1
DTH: Delayed-type Hypersensitivity
ELISPOT: Enzyme-Linked Immunospot
FACS: Fluorescence-Activated Cell Sorting
FDA: Food and Drug Administration
GD2: Disialoganglioside
GM-CSF: Granulocyte-Macrophage Colony-Stimulating Factor
GMP: Good Manufacturing Practice
HER2: Human epidermal growth factor receptor 2
HLA: Human Leukocyte Antigen
ICANS: Immune-effector Cell-Associated Neurotoxicity
ICI: Immune Checkpoint Inhibition
IDO: 3-Idolamine-Dioxygenase
IFN-γ: Interferon-γ
IL-4: Interleukin-4
IL13Rα2: Interleukin-13 receptor alpha 2
JAK-STAT: Janus kinase-signal transducer and activator of transcription
KIR: Killer-Cell Immunoglobulin-like Receptor
LAG-3: Lymphocyte-Activation Gene 3
MACS: Magnetic-Activated Cell Sorting
MAGE: Melanoma Antigen Gene
MAPK: Mitogen-Activated Protein Kinase
MART-1: Melanoma Antigen Recognized by T cells 1
MEK: Mitogen-activated protein kinase
MICA: induced self-ligands including HLA class I chain-related protein A
MICB: HLA class I chain-related protein B
MRI: Magnetic Resonance Imaging
NK: Natural Killer
NRAS: Neuroblastoma RAS viral oncogene homolog
NY-ESO-1: New York Esophageal Squamous Cell Carcinoma 1
ORR: Objective Response Rates
FoxP3: Forkhead box P3
PD-1: Programmed Cell Death Protein 1
PD-L1: Programmed Death Ligand 1
PFS: Progression-Free Survival
PGE2: Prostaglandin E2
PR: Partial Responses
PRAME: Preferentially Expressed Antigen in Melanoma
TCR: T-Cell Receptor
TCR-T: T-Cell Receptor-engineered T cells
TH1: T helper type 1
TIGIT: T cell immunoreceptor with Ig and ITIM domains
TIL: Tumor-Infiltrating Lymphocyte
TIME: Tumor Microenvironment
TLR: Toll-Like Receptor
TNF-α: Tumor Necrosis Factor α
TRAIL: TNF-Related Apoptosis-Inducing Ligand
TYRP1: Tyrosinase-Related Rrotein 1
ULBP: UL16-Binding Protein
UV: Ultraviolet
